# The pathophysiology of osteoporosis in obesity and type 2 diabetes in aging women and men: The mechanisms and roles of increased bone marrow adiposity

**DOI:** 10.3389/fendo.2022.981487

**Published:** 2022-09-15

**Authors:** Dalia Ali, Michaela Tencerova, Florence Figeac, Moustapha Kassem, Abbas Jafari

**Affiliations:** ^1^ Department of Molecular Endocrinology, KMEB, University of Southern Denmark and Odense University Hospital, Odense, Denmark; ^2^ Laboratory of Molecular Physiology of Bone, Institute of Physiology of the Czech Academy of Sciences, Prague, Czechia; ^3^ Department of Cellular and Molecular Medicine, University of Copenhagen, Copenhagen, Denmark

**Keywords:** aging, osteoporosis, bone marrow adiposity, bone fragility, type 2 diabetes (T2D), obesity

## Abstract

Osteoporosis is defined as a systemic skeletal disease characterized by decreased bone mass and micro-architectural deterioration leading to increased fracture risk. Osteoporosis incidence increases with age in both post-menopausal women and aging men. Among other important contributing factors to bone fragility observed in osteoporosis, that also affect the elderly population, are metabolic disturbances observed in obesity and Type 2 Diabetes (T2D). These metabolic complications are associated with impaired bone homeostasis and a higher fracture risk. Expansion of the Bone Marrow Adipose Tissue (BMAT), at the expense of decreased bone formation, is thought to be one of the key pathogenic mechanisms underlying osteoporosis and bone fragility in obesity and T2D. Our review provides a summary of mechanisms behind increased Bone Marrow Adiposity (BMA) during aging and highlights the pre-clinical and clinical studies connecting obesity and T2D, to BMA and bone fragility in aging osteoporotic women and men.

## Osteoporosis

Osteoporosis is a chronic-skeletal disorder characterized by low bone mass, and deterioration in the microarchitecture of the bone tissue that results in bone fragility and increased fracture risk (1). It is the most common bone disease worldwide, affecting more than 200 million people and causing more than 9 million fractures in the year 2000 (2). In 2016, a European study estimated that 6.6% of men and 22.1% of women over the age of 50 years were diagnosed with osteoporosis, and around 3.5 million with fragility fractures. Osteoporosis causes vertebral and hip fractures, as well as chronic pain, that in many cases, are associated with disability and reduced life quality. Fractures, due to osteoporosis, require hospitalization and increase risk of mortality by 20%, and in 50% of the cases, result in chronic disability (3). The World Health Organization (WHO) has defined osteoporosis as a disease of low bone mass as measured by Dual-Energy X-ray Absorptiometry (DEXA) with Bone Mineral Density (BMD) equal or less than -2.5 standard deviations of the average value for young healthy persons (known as (T-score≤-2.5) (4, 5).

## Pathophysiology of bone loss in age-related osteoporosis

Osteoporosis is primarily attributed to aging and sex-steroid deficiency, which at the cellular level leads to increased bone resorption by osteoclasts and decreased bone formation by osteoblasts (6, 7). A number of underlying pathogenic mechanisms of osteoporosis have been proposed, such as estrogen deficiency-associated Bone Marrow (BM) inflammation (8) or decreased levels of anabolic hormones (9, 10). However, increasing evidence has suggested a possible role of Bone Marrow Adiposity (BMA) in pathogenesis of bone loss in osteoporosis.

## BMA and bone loss in age-related osteoporosis

Aging is defined as a gradual loss of normal tissue homeostasis and progressive deterioration of the organ functions, due to accumulation of cellular/DNA damage and senescence throughout aging (11). Aging is associated with significant bone loss and structural bone damage, due to accelerated trabecular thinning and disconnection, cortical thinning, and porosity (12), leading to increased fracture risk. Thus, osteoporosis is an exaggerated expression of aging process in the bone tissue (13). One of the key underlying mechanisms of bone loss in age-related osteoporosis is the altered lineage allocation of Bone Marrow Stromal Cells (BMSCs), associated with enhanced differentiation towards adipocyte lineage, leading to expansion of the Bone Marrow Adipose Tissue (BMAT), at the expense of compromised osteoblast differentiation and bone formation (13). Comprehensive characterization of the epigenomic and transcriptional changes associated with osteoblast and adipocyte differentiation of BMSCs has shown that the inverse correlation between fate specification of BMSCs into osteoblast versus adipocyte lineages is regulated by a large and diverse network of transcription factors (14).

Expansion of BMAT is caused by the increase in adipocyte size and/or number and is defined as the proportion of the BM cavity occupied by the adipocytes (15). The expansion of BMAT in aging can lead to space limitation for other cells that are required for normal skeletal homeostasis, such as BMSCs, osteoblastic, or hematopoietic cells (16). In addition, factors that are secreted by Bone Marrow Adipocytes (BMAds) can also contribute to altered skeletal homeostasis after BMAT expansion, such as adipokines, RANKL, immune-regulatory and pro-inflammatory cytokines. Examples of BMAd-secreted factors that can regulate bone cell function include RANKL, adiponectin, leptin, legumain, and chemerin (17–19).

Age-related expansion of BMAT and osteoporotic bone loss are mediated through intrinsic and extrinsic mechanisms, such as cellular senescence within the bone microenvironment or age-related endocrine dysfunction.

## Intrinsic mechanisms

### Accumulation of senescent cells

Aging is associated with accumulation of senescent cells, due to diverse stress stimuli (e.g. shortening of telomeres, oncogenic or metabolic insults), causing the cell to enter a state of irreversible growth arrest (20). Senescence is also associated with different cellular alterations including changes in chromatin organization, gene expression (e.g. increased expression of cell cycle regulators/tumor suppressors such as p16^Ink4a^ and p53), mitochondrial dysfunction, and resistance to apoptosis, reviewed in (21, 22). In addition, a key feature of senescent cells is development of a distinctive secretome, known as Senescence-Associated Secretory Phenotype (SASP), characterized by secreting high levels of pro-inflammatory cytokines, immune modulators, growth factors, and proteases, that can spread throughout the tissue, and further exacerbate the senescence and tissue dysfunction (23–29).

Murine studies using the SAMP6 model had shown that accelerated senescence is associated with enhanced adipogenesis and inhibited osteoblastogenesis within the bone microenvironment, leading to compromised bone formation and decreased bone mass (30). Analysis of human bone specimens had shown presence of senescence microenvironment in the bone tissue obtained from old individuals compared to young control subjects, evidenced by increased expression of p16^Ink4a^ and p21 (31). Furthermore genetic depletion of senescent cells, using the inducible INK-ATTAC ‘suicide’ transgene that eliminates the p16^Ink4a^ expressing cells, in a murine pre-clinical model of age-related osteoporosis, exhibited anti-resorptive and anabolic impact on bone, leading to decreased BMAT and increased bone mass (32).

Aging is also accompanied with increased levels of Reactive Oxygen Species (ROS) and oxidative stress in the bone microenvironment, leading to impaired osteoblast differentiation and function, and enhanced adipocyte differentiation (33). It was shown that presence of the antioxidant agent Resveratrol in the cultures of human BMSCs obtained from old individuals enhances differentiation and function of osteoblasts and inhibits formation of BMAds (34). In addition, *in vivo* murine studies using irradiation or physiological aging models, in which increased levels of ROS within the bone microenvironment are associated with expansion of BMAT and compromised skeletal homeostasis, indicated that antioxidant agents Dasatinib and Quercetin reduced BMAT and enhanced bone formation, by decreasing the ROS levels and senescent cells (35). These studies provide strong evidence for use of antioxidants as a promising approach for preventing age-related bone loss and osteoporosis.

Few studies have reported that senescence can be regulated *via* transcription factors (36, 37). Forkhead box rotein P1 (FOXP1) is involved in transcriptional control of BMSC senescence, and its expression levels had been shown to decline with aging, and inversely correlate with p16^Ink4a^ expression. Mouse genetic studies had shown that conditional depletion of Foxp1 in BMSCs leads to premature aging, bone loss, and increased BMA *via* Foxp1 interactions with different members of C/EBP family proteins, such as C/EBP β/δ, which are key modulators of adipogenesis (38). Another study in aged mice revealed that senescence is associated with down regulation of expression of osteoblast transcription factors such as Runx2 and Dlx5, leading to impaired osteoblastogenesis and enhanced adipogenesis of BMSCs *via* up-regulation of adipocyte-specific transcription factor PPAR-γ (39). Furthermore, an *in vivo* study using murine irradiation model indicated the increased burden of senescence in bone cells from day 1 to day 7 after irradiation (evidenced by increased expression of p21), followed by expansion of BMAT starting at day 7 and continuing until day 42 post irradiation (35). This study also indicated a direct correlation between the increased senescence burden in the bone tissue and up-regulation of mir-27a, which is known to be involved in obesity and regulation of adipose-tissue related transcription factors (40). These studies provide strong evidence for role of senescent microenvironment in expansion of BMAT.

### DNA damage

DNA damage can be caused by exogenous factors such as Ionizing Radiation (IR), chemotherapeutic agents, and Ultraviolet (UV) light exposure, as well as endogenous factors such as ROS that are generated by mitochondria in the process of ATP production (41). These factors have been shown to cause impairment of osteoblastogenesis, and enhanced adipogenesis within the bone microenvironment. Pre-clinical and clinical studies have shown that cancer treatment using IR or chemotherapy leads to significant expansion of BMAT, which may contribute to the progressive bone loss in cancer survivors (42–44). In addition, it is shown that aging is associated with an intrinsic defect in osteoblasts, due to accumulation of DNA damage, leading to decreased osteoblast number, compromised osteoblast function, and induction of osteoclast formation (45). This study employed young (6 months) and old (20-24months) Osx1-Cre; TdRFP-mice and showed that the number of TdRFP-Osx1 cells are decreased by 50% in the BM of old male and female mice as compared to young mice. The TdRFP-Osx1 cells obtained from old mice also exhibited increased levels of DNA damage and senescence markers, such as formation of γ-H2AX foci, phosphorylation of p53, and G1 cell cycle arrest. BMSCs obtained from old mice also exhibited increased expression of SASP markers, leading to increased osteoclast formation, as well as increased expression of the adipogenic transcription factor PPAR-γ (45).

Murine studies using radiation-induced osteoporosis had indicated that accumulation of DNA damage leads to significant increase in the percentage of adipocytes within the BM. In addition, proteosome inhibitors or sclerostin neutralizing antibody reduced the BMA and prevented the trabecular bone structural deterioration post-radiation, *via* induction of DNA-repair (at least partially) (46, 47).

Although these studies provide evidence for the role of DNA damage in expansion of BMAT, the exact underlying mechanisms are not fully understood. However, induction of senescence (35) and the altered expression of genes involved in regulating the fate specification of BMSCs toward osteoblast and adipocyte lineages are among possible mechanisms. In addition, these studies suggest that mitigation of DNA damage can be employed as an approach for reducing BMA and improving the bone architecture post radiation.

## Extrinsic mechanisms involved in age-related bone loss and BMAT expansion

Extrinsic factors such as disrupted hormonal status, malnutrition, and reduced physical activity, have an important role in disturbed skeletal homeostasis in aging, leading to inhibited bone formation and enhanced bone resorption (48).

### Endocrine Aging

Bone growth and maintenance of the skeleton is regulated *via* different endocrine factors (hormones) such as estrogens and androgens (49, 50). Fuller Albright proposed 70 years ago, that osteoporosis in women was caused by estrogen deficiency (51) and that bone loss induced *via* ovariectomy in women can be reversed by estrogen therapy (52). Estrogen is involved in molecular signaling in bone and regulation of BMAds (53–55) as estrogen is a positive regulator of osteogenesis through activation of estrogen receptor (ER)-dependent cytoplasmic kinases, BMPs and Wnt signaling (56, 57).

Testosterone exhibits an anabolic impact on bone formation in the periosteal surface in mice (58), and that oral administration of testosterone reduces the accelerated bone loss in orchiectomized mice (59). Gradual reduction in the testosterone levels in aging men are associated with reduction in bone mass (60, 61), and osteoblast dysfunction (62). In a clinical study of 350 men between the ages of 20-90 years, the levels of bioavailable testosterone were decreased by 64%, and bioavailable estrogen by 47% in old versus young individuals. This study also indicated that age-related bone loss in men is associated with decline in testosterone as well as estrogen (63). Another human study investigated the impact of endogenous estrogen and testosterone production on bone, using 59 elderly men and demonstrated that estrogen is the dominant sex-steroid hormone protecting men from age-related bone resorption, whereas both hormones of estrogen and testosterone are critical for bone formation (64).

Several mechanisms have been proposed to be involved in mediating the direct/indirect roles of estrogen deficiency in accelerated bone loss. Estrogen deficiency is involved in increased expression of pro-inflammatory cytokines in the bone microenvironment such as TNFα, IL1, IL-6 and receptor activator of RANKL/OPG/RANK system that regulates osteoclast differentiation (65–67). Estrogen deficiency also leads to decreased production of endogenous antioxidants (68, 69), which together with the elevated ROS production and the senescence microenvironment observed during aging or in obese mice and human, can lead to significantly increased levels of oxidative stress in the bone microenvironment (70, 71).

Studies using ovariectomized rats and postmenopausal women revealed increased bone turnover in response to estrogen deficiency, i.e. increased bone resorption and bone formation, manifested by increased number of osteoblast precursors, osteoblast proliferation and increased osteoblast number (72, 73). However, the increased bone formation levels are not enough to account for the bone loss due to elevated bone resorption, thereby leading to bone loss.

An interesting question that has been raised is whether the biological sex has an impact on the expansion of BMAT during aging? Griffith et al, investigated BMD and BMAT in the lumbar spine of 259 healthy subjects (145 females, 114 males; age range: 62-90 years) using MR spectroscopy of L3 vertebral body, and revealed that in males, BMAT increases gradually throughout life, whereas in females, BMAT increased between 55 and 65 years (74). BMAT in the vertebral bones in females with the age of more than 60 years increased by nearly 10% higher compared to age-matched males, indicating the positive impact of estrogen deficiency on BMAT expansion. In addition, human studies have shown that aging and loss of steroid hormones make women more vulnerable to the negative impacts of BMAT on loss of trabecular bone at the spine and femoral neck, and greater loss of spine strength (75), while in men vertebral BMAT is significantly increased with osteoporosis (76). **Table 1** provides a summary of several clinical and preclinical studies related to association of endocrine aging and BMAT.

**Table 1:** Summary of several clinical and preclinical studies related to investigating the association of endocrine aging and BMAT expansion .

Study	Design	Outcome	Reference
The Effect of Roux-en-Y Gastric Bypass on Bone Marrow Adipose Tissue and Bone Mineral Density in Postmenopausal, Nondiabetic Women	14 postmenopausal, nondiabetic obese women were scheduled for laparoscopic Roux-en-Y gastric bypass surgery (RYGB).	Decrease in BMAT at the level of the L3-L5 vertebrae, 12 months post-surgery measured by quantitative chemical shift imaging (QCSI) with magnetic resonance imaging (MRI), as well as decrease in vertebral volumetric BMD (vBMD).	(77)
Short-Term Effect of Estrogen on Human Bone Marrow Fat.	Measured vertebral bone marrow fat fraction every week for 6 consecutive weeks in 6 postmenopausal women before, during, and after 2 weeks of oral 17-β estradiol treatment (2 mg/day).	17-β estradiol rapidly reduced the marrow fat fraction, suggesting that 17-β estradiol regulates bone marrow fat independent of bone mass.	(78)
Effects of estrogen therapy on bone marrow adipocytes in postmenopausal osteoporotic women	bone biopsies from a randomized, placebo-controlled trial involving 56 postmenopausal osteoporotic women (mean age, 64 years) treated either with placebo (PL, n = 27) or transdermal estradiol (0.1 mg/d, n = 29) for 1 year.	AV/TV and BMAd number increased in the PL group but were unchanged (BMAd) or decreased in the E group. E treatment also prevented increases in mean adipocyte size over 1 year. Increased bone loss and bone marrow adipocyte number and size in postmenopausal osteoporotic women may be due estrogen deficiency.	(79)
Association of vertebral bone marrow fat a with trabecular BMD and vertebral fracture in older adults	257 participants, mean age was 79 years,	Vertebral BMA was associated with lower BMD and vertebral fractures in older women.	(80)
Correlation of vertebral bone marrow fat content with abdominal adipose tissue, lumbar spine, bone mineral density, and blood biomarkers in women with type 2 diabetes mellitus	Thirteen postmenopausal women with T2D	There is a correlation between BMA and subcutaneous adipose tissue in women with and without T2D and with visceral adipose in women with T2D.	(81)
Bone Marrow Adiposity and prediction ofBone Loss in Older Women	women (n = 148) and mean age (80.9 ± 4.2) years	BMA is associated with higher loss of trabecular bone at the spine area and femoral neck, and greater loss of spine strength	(75)
Changes in BMA during aging in males and females	145 females, 114 males; age range (62-90) years.	Marrow fat content increases significantly in female subjects of age range (55 and 65) years of age while male subjects increase in marrow fat at a steady rate. Females aged older than 60 years have a higher marrow fat content than males.	(74)
Effects of risedronate on bone marrow adipocytes in postmenopausal women	Transiliac bone biopsies from a randomized, placebo-controlled clinical trial in women with postmenopausal osteoporosis (n=14 per group)	Risedronate reduced age-dependent expansion of BMAT, compared to placebo.	(82)
Analysis of vertebral bone mineral density, marrow perfusion, and fat content in healthy men and men with osteoporosis using dynamic contrast-enhanced MR imaging and MR spectroscopy	MR imaging of the lumbar spine in 90 men (mean age, 73 years; range, 67-101 years)	Increased BMA in osteoporotic patients compared to osteopenic subjects. Increased BMA in osteopenic subjects compared to healthy control individuals.	(76)
Effect of estrogens on bone marrow adipogenesis and Sirt1 in aging C57BL/6J mice	Young skeletally mature (5 months) and old (22–24 months) female C57BL/6J mice were either gonadally intact, OVX or OVX +E2	Significant decreasing effect of E2 on BMAT in both young and old mice.	(83)
Analysis of bone marrow fat content in relation to bone remodeling and serum chemistry in intact and ovariectomized dogs	Beagle dogs (6 control, 9 ovariectomized)	BMAT was expanded (11 months post ovariectomy) together with reduced hematopoietic volume fraction, associated with decrease in estrogen levels.	(84)

Another question that has been raised recently is whether gender-affirming interventions have an impact on bone microstructure and BMA?

Gender-affirming interventions using hormone therapy or surgery aim to align the physical characteristics with an individual’s gender identity (85). Bretherton et al. recently employed high-resolution peripheral quantitative computed tomography of the distal radial and tibial microarchitecture and showed that trans men have normal bone microarchitecture as compared to cis female controls, whereas trans women had deteriorated bone microarchitecture compared to cis male controls (86). In addition, Nasomyont et al. showed lower bone mass acquisition and greater increases in BMAT indices in Transgender and Gender Non-Conforming (TGNC) youth after 12 months of pubertal suppression with gonadotropin-releasing hormone agonists (87). Additional investigations are required to establish the impacts of gender-affirming interventions on bone and BMA, and the related underlying mechanisms, also in the context of aging, obesity, and diabetes.

## Obesity and diabetes

### Bone fragility and microstructural changes in BM associated with T2D

Aging, osteoporosis and metabolic diseases such as obesity and diabetes robustly affect microstructural changes in the BM and contribute to impairment of bone homeostasis, which leads to higher risk of bone fractures (88, 89). The altered cellular landscape and molecular networks within the bone microenvironment in obesity and diabetes induce changes in trabecular and cortical bone volume or increased amount of BMAT, leading to a detrimental effect on bone quality and strength.

Osteoporosis and aging are characterized with decreased BMD, along with increased BMAT and accompanied by increased resorption activity in the BM in mice and humans (90, 91). Metabolic complications associated with impairment of glucose homeostasis have been shown to contribute to higher risk of bone fractures and accelerate the manifestation of bone fragility and osteoporosis. Previous studies in mice and humans under High Fat Diet (HFD) condition have reported increased BMAT formation evaluated by measurement of BMAT volume, BMAd size and number (70, 92–98), which was correlated with increased, unchanged, or decreased BMD in mice depending on the employed diabetic model (T1D or T2D) (70, 71, 94, 95, 99), but associated with a higher risk of fractures. Therefore, recent studies have focused on the design of experiments to include measurement of BMAT parameters in relation to bone fragility. Indeed, our recent study using T2D diabetic animal model (HFD in combination with streptozotocin) confirmed the expansion of BMAT in T2D mice along with decreased bone volume, which contributed to the delayed bone healing in monocortical fracture model (100).

Interestingly in humans, obesity is associated with increased BMD in children and adults (89, 101, 102). Along with this finding, several clinical studies including ours have noted reduced bone turnover in patients with metabolic diseases (71, 103). Paradoxically, there are also clinical studies suggesting that women and men with high BMI and T2D may be protected from osteoporosis, due to increased BMD (104–109). These clinical studies are summarized in **Table 2**. The mechanisms underlying the higher bone mass but reduced bone turnover in patients with obesity and T2D were partially explained by our clinical study in obese subjects that revealed hypermetabolic status of BMSCs and accelerated senescent BM microenvironment contributing to the bone fragility (71). These changes lead to impairment of the bone material properties and increased cortical porosity in T2D (126). However, further follow up studies are needed to collect more clinical data on bone microstructural parameters along with BMAT volume in relation to bone quality in specific target groups of patients, to better predict the fracture risk.

**Table 2:** Clinical studies investigating effect of obesity and diabetes on bone in men & women.

Study	Type of study	Participants	Main outcome	Reference
Relation between body size and bone mineral density in elderly men and women	Cross-sectional	1492 ambulatory white adults, 55–84 years	High BMI was positively related with high BMD. the mechanical effect of weight increased BMD.	(110)
Are general obesity and visceral adiposity in men linked to reduced bone mineral content resulting from normal ageing? A population-based study	RCT	Polish men (272 men, 20–60 years)	Visceral adiposity (assessed by waist/hip ratio) was associated with reduced bone mass in men.	(111)
Associations between components of the metabolic syndrome versus bone mineral density and vertebral fractures in patients with type 2 diabetes	RCT	187 men (28–83 years) and 125 postmenopausal women (46–82 years) with type 2 diabetes	Obesity and diabetes were associated with increased femoral neck bone mineral density. Effects on fracture risk were site dependant.	(112)
Is obesity protective for osteoporosis? Evaluation of bone mineral density in individuals with high body mass index	RCT	398 patients (291 women, 107 men, age 44.1 + 14.2 years, BMI 35.8 + 5.9 kg/m^2^	Obesity had a negative impact on lumbar BMD than expected for that age.	(113)
Bone mineral density of the spine in normal Japanese subjects using dual-energy X-ray absorptiometry: effect of obesity and menopausal status	RCT	N= (1,048 women, age 40-49 and >50) Menopause N= (248 men, age 20-29 and >50)	Bone mineral density measurements at the lumbar spine using DEXA-scan revealed that bone loss starts at early menopause stage and concluded a positive correlation between obesity and BMD, particularly in postmenopausal women.	(114)
Determinants of total body and regional bone mineral density in normal postmenopausal women–a key role for fat mass	RCT	N= (140 post-menopausal women) (age= 45-71 years, mean 58years)	Total body BMD was positively related to fat mass, and similar relationships were found in other body regions as in the lumbar spine and proximal femur.	(115)
Obesity and Postmenopausal Bone Loss: The Influence of Obesity on Vertebral Density and Bone Turnover in Postmenopausal Women.	Cross-sectional	N= (176 women aged 45-71 years). (49 perimenopausal) (28 obese peri-menopausal) (49 obese post-menopausal) Mean age was the same yet there was significant change in the weight.	In non-obese post-menopausal women, BMD was lower, and higher serum osteocalcin (OC) and fasting urinary calcium to creatinine (Ca : Cr). Obesity may be protective in post-menopause state.	(106)
Influence of obesity on bone density in postmenopausal women	Case-control	N= (588 women) Age = (41 to 60 years) Group 1: (1-6 years since menopause) Group 2: (6-10 years since menopause)	Positive influence of obesity at increasing BMD at lumbar spine, femoral neck (FN), and trochanter (TR) between the groups, yet the role of obesity is demolished by the impact of estrogen deficiency and aging.	(116)
Cigarette Smoking, Obesity, and Bone Mass	Case-control	N= (84 healthy, peri- and postmenopausal women) were studied prospectively over 3.5 years.	Menopause combined with obesity led to bone loss, independent of smoking.	(117)
Plasma Leptin Values in Relation to Bone Mass and Density and to Dynamic Biochemical Markers of Bone Resorption and Formation in Postmenopausal Women	Case-control	N= (54 post-menopausal women)	Positive correlation of leptin plasma levels with body weight, fat mass and BMD yet no correlation with biochemical markers of either osteoclastic or osteoblastic activity.	(118)
Calcium Supplementation Suppresses Bone Turnover During Weight Reduction in Postmenopausal Women	Randomized-double blind placebo control	N= (43 post-menopause women) Subject to take calcium citrate 1gm/day (N= 21), subjects to take placebo (N=22).	Obesity in postmenopausal women tend to increase MD and that weight loss in postmenopausal women should consume calcium supplement (1500 mg/day) to prevent a high rate of bone turnover and loss in BMD.	(119)
Factors affecting bone mineral density in postmenopausal women	Cross-sectional	N= (537 women) Age (of 67.9 ± 6.7 years and mean menopause duration (MD) of 15.8 ± 5.1 years)	Obesity may protect again osteoporosis as its associated with higher BMD also significant positive association between osteoporosis and menopausal duration.	(120)
Influence of obesity on vertebral fracture prevalence and vitamin D status in postmenopausal women	RCT	N= (429 post-menopausal women (mean age, weight and BMI of 59.5 ± 8.3 (50 to 83) years, 75.8 ± 13.3 (35 to 165) kgs and 29.9 ± 5.2 (14.6 to 50.8) kg/m^2^)	Obesity was correlated with increased BMD yet vertebral fractures were related to duration of menopause, low vitamin D intake and increased osteoporosis.	(121)
Relationship between body composition, body mass index and bone mineral density in a large population of normal, osteopenic and osteoporotic women	RCT	N= (6,249 Italian women, aged 30–80 years)	Obesity was increased with age yet is believed to be protective against osteoporosis as BMD is increased, yet obesity did not decrease the risk of osteopenia, with aging above 50years, the risk of osteopenia and osteoporosis is increased, respectively.	(122)
Evaluation of bone loss in diabetic postmenopausal women	Cross-sectional	N= (200 diabetic postmenopausal women with 400 non-diabetic postmenopausal women) Age (65.23 ± 4.80 non-diabetic vs. 66.91 ± 5.78 years in diabetic)	Diabetes increases the risk of osteopenia and osteoporosis when comparing postmenopausal diabetic and no-diabetic women.	(123)
Influence of obesity on bone mineral density in postmenopausal asthma patients undergoing treatment with inhaled corticosteroids	Case-control	N= (46 patients with asthma taking inhalations of **corticosteroids**, age 62.5 ± 10.6 and 60 healthy female controls, age 63.0 ± 6.1) all post-menopaused.	Obesity in asthmatic patient is positively correlated with decreased osteoporosis yet this effect is overcome by aging and years since menopause.	(124)
Obesity Is Not Protective against Fracture in Postmenopausal Women: GLOW	RCT	N= (60,393 women aged >55 years) menopause women.	Obesity is not protective against fracture risk in ankle and upper leg was significantly higher in obese than in nonobese women.	(125)

Human studies had shown that expansion of visceral fat is associated with enhanced adipocyte formation in the BM microenvironment in osteoporotic obese women (97). Furthermore, BMAT was shown to be expanded in the bone biopsies from overweight and obese subjects compared to healthy age-matched individuals (127). Similarly, BMSCs obtained from obese men exhibited enhanced adipocyte differentiation and accelerated senescence phenotype that would contribute to the skeletal fragility in obesity (71). T2D is another risk factor in skeletal fragility and osteoporosis in aging men and postmenopausal women (128–130), despite the increase in BMD or independent of BMD (131, 132). The combination of obesity and T2D were shown to exhibit higher serum insulin levels and BMA at the lumbar spine and femoral metaphysis compared to the subjects without T2D. In addition, it is shown that lumbar spine BMD is inversely associated with the lumbar adiposity in adults with morbid obesity, and that morbid obesity and T2D exhibits a higher BMA than non-diabetic controls of similar weight (98). In agreement with these observations, Sheu et al. reported that in 38 old diabetic men there was a higher vertebral BMA, spine and hip BMD and a higher fracture risk when compared to control men without diabetes (133).

Fazeli et al. examined how human BMAT responds to acute nutrient changes by employing 10 days of high calorie protocol followed by 10 days fasting protocol. This study indicated a significant increase in vertebral BMAT after high calorie feeding and fasting, and that high calorie feeding up-regulated the inflammatory marker TNFα in BMAds, which was decreased upon fasting (134).

Kim et al., reported that BMAT levels were reduced after bariatric surgery in obese T2D patients compared to the obese non-diabetic individuals, and that there was an inverse correlation between the changes in BMAT in lumbar spine and femoral neck and spine BMD (135). In addition, 6 weeks of low calorie diet was reported to induce weight loss in obese T2D subjects (19 women and 10 men) together with decrease in vertebral BMA (136).

Pre-clinical studies using murine models have also provided evidence for impact of obesity and diabetes on BMAT and bone mass. HFD-induced-obesity using C57Bl/6J male mice, was reported to lead to enhanced adipocyte differentiation of BMSCs, associated with increased *in vivo* BMAT volume and decreased trabecular and cortical bone mass (70). Another *in vivo* study in male C57Bl/6J mice reported that HFD-induced obesity and T2D compromised the skeletal macro- and micro-architecture of the bone (137). In addition, increased bone resorption is also shown to contribute to the reduced bone mass and strength in a pre-clinical obese-diabetic mouse model (138).

Transition of metabolic status from obesity to insulin resistance and T2D is accompanied with changes in glucose, insulin and circulating lipid levels, as well as changes in inflammatory pathways and hormonal alterations. The relative contribution of each of these factors to the expansion of BMAT, compromised bone quality, and skeletal fragility remain unclear. In addition, the length of exposure to obesogenic and diabetic conditions has an impact on the effect of these conditions on the bone microstructure in mice and humans. Therefore, it is important to include more longitudinal studies to investigate the changes of bone and BMAT parameters measured at several timepoints, in order to better understand the impact of different elements of obesity and T2D on the bone quality and strength and to determine the parameters that can be employed for predicting the risk of fracture.

### Role of BMSC dysfunction and BMAds in pathogenesis of increased bone fragility in obesity and T2D

Although different mechanisms have been proposed to be involved in pathogenesis of bone fragility in obesity and T2D, the underlying cellular and molecular events are not yet fully understood. Impaired BMSC function and differentiation is one of the key mechanisms that is suggested to have role in expansion of BMAT and decreased bone mass and quality in obesity and T2D, thereby leading to increased skeletal fragility during aging.

Increased levels of oxidative stress and senescence within the bone microenvironment in obesity and T2D are among factors that can contribute to BMSC dysfunction, and a shift in BMSC differentiation phenotype, favoring adipogenesis than osteogenesis (139–142).

BMSCs from obese subjects are shown to exhibit a hypermetabolic state and a shift of molecular phenotype towards adipocyte progenitors, together with altered expression of genes involved in metabolic regulation, such as glycolysis and oxidoreductase activity. The hyper-metabolic state of BMSCs in obesity is associated with increased abundance of leptin receptor and insulin receptor positive cells and an accelerated senescence phenotype and increased ROS production (71). Leptin and leptin receptor signaling promotes adipogenesis in the BM, in response to HFD (143). In addition, insulin receptor signaling is reported to be increased under oxidative stress condition, associated with cellular senescence phenotype (144).

The above-mentioned studies provide evidence for the role of cell autonomous defects in BMSCs in pathogenesis of bone fragility in obesity and T2D. However, Devlin et al. employed the male TALLYHO/JngJ murine model of T2D and indicated that in the context of early onset T2D, impaired bone formation and the consequent skeletal deficits are due to the altered bone microenvironment, but not the cell autonomous defects in BMSCs (145).

Increasing evidence has shown that BMAds may also play an important role in altered bone microenvironment in obesity and T2D (146–148). BMAds secrete a number of adipokines and cytokines that affect bone cell functions directly or indirectly, e.g. through modulation of inflammation within the bone microenvironment (149).

IL-6 is one of the cytokines that is secreted by BMAds and increases osteoclast formation and bone resorption, thereby leading to decreased BMD in men and women (150, 151). IL-6 enhances osteoclastogenesis by stimulating the expression of RANKL on stromal/osteoblastic cells (152), and also by direct support of osteoclast formation through RANKL-independent mechanisms (153).In addition, Li et al. revealed that IL-6 KO mice are protected against HFD-induced trabecular bone loss, and the associated BMSC senescence and BMAT expansion (154). BMAd-derived factors that can affect skeletal homeostasis are reviewed by Aaron et al. (155).

### Can the deleterious effects of obesity and T2D on the skeleton be accelerated by postmenopausal estrogen deficiency?

In post-menopausal women, obesity is another accompanying complication besides estrogen deficiency that affects energy homeostasis and metabolism of bone and adipose tissue (156). Menopause is associated with shift in BMSCs phenotype towards adipogenesis, reduced osteogenesis, increase in the osteoporotic bone phenotype and fracture risk (157–160). Possible mechanisms of deleterious effects of estrogen deficiency on bone metabolism is discussed above (Endocrine aging and **Table 1**). Combination of estrogen deficiency and obesity includes a chain of pathologic events, leadingto disturbed skeletal homeostasis that further contribute to the accelerated aging of BMSCs and compromised bone regeneration and increased fracture risk (161). Several clinical studies in postmenopausal women (presented in **Table 2**) have however shown that obese postmenopausal women exhibit increased BMD, that is paradoxically associated with increased bone fragility and fracture risk, whereas the status of BMA is not evaluated in these studies (116, 162).

## Interventions to reduce BMA, enhance bone mass, and reduce bone fragility

To reverse the detrimental effects of metabolic complications on bone fragility, various interventions have been applied including diet, exercise, or pharmacological treatment. However, in many of these studies there is limited information on BMAT changes in relation to bone and energy metabolism, especially in human studies, as the evaluation of BMAT (fat content in BM) is not a common clinical practice.

### Nutritional intervention

A “Bone Healthy Diet” should contain the nutritional components that are required for normal skeletal homeostasis, such as calcium, vitamin D, vitamin C, vitamin K, vitamin A, protein, and phytoestrogens, that help enhancing BMD and reduce bone loss (163).

1,25 dihydroxy vitamin D is the active form of vitamin D, and its main function in bone is to modulate osteoblast proliferation, differentiation, and providing suitable microenvironment for bone mineralization. Presence of calcitriol in aging osteoblast cultures revealed enhanced osteoblastic function (164). Different mechanisms of action have been proposed for the effect of vitamin D on osteoblast differentiation and bone formation (165, 166). Many studies have revealed the significance of Vitamin D deficiency in inducing bone loss, osteoporosis, and affecting the function of skeletal muscle. It is shown that vitamin D restricted diet (for 28 days) leads to decreased bone mineral content and muscle mass in ovariectomized rats challenged with HFD (167). Vitamin D deficiency in the elderly people causes secondary hyperparathyroidism and increased risk of hip fracture (168), as low circulating levels of vitamin D increases parathyroid hormone (PTH) levels, which induce bone resorption and bone loss in elderly subjects (169–171). Vitamin D combined with calcium is also used to prevent osteoporosis (172).

### Is there a connection between vitamin D and BMA regulation?

Calcitriol has shown anti-adipogenic effects in cultures of 3T3-L1 cells (173, 174) as it inhibits the transactivation capacity of PPARγ, through Vitamin D Receptor (VDR) signaling. VDR could inhibit PPARγ transactivation activity, by competing for binding to their common heterodimer partner RXR (175). In VDR null mice, the BMSCs exhibited high expression of PPARγ, along with higher expression of DKK1 and SFRP2, that are inhibitors of the pro-osteogenic canonical Wnt signaling pathway, and the expression of these Wnt inhibitors was downregulated by calcitriol in wild-type BMSCs, in the absence or presence of adipogenic inducers (176). Similar results were observed in human BMSCs (177).

Anti-adipogenic effect of vitamin D is also observed in femur-derived mouse BMSCs and is mediated through inhibiting the expression of aP2 and adipsin (178). Vitamin D3 supplementation is recommended upon aging (179) and leads to increasing the circulating IGF-1 levels (180). Increasing the levels of circulating IGF-1 may help in counteracting the age associated BMAT expansion (181, 182). Thus, these *in vitro* and *in vivo* studies suggest that vitamin D signaling may contribute to the regulation of BMA. However, more clinical investigations are required to directly examine the effect of vitamin D supplementation on BMA in human, and to assess the correlation between vitamin D levels, BMAT volume, and fracture risk.

### Lifestyle factors

Creating awareness and understanding of the lifestyle factors that may delay the aging bone phenotype is crucial. One of the most important lifestyle factors is the physical activity (114). Physical activity leads to strengthening of the hip and spine due to skeletal loading and inducing bone formation at the stressed skeletal sites (183).

Styner et al. employed a murine model (4 weeks old female C57BL/6 mice) to investigate the impact of running exercise on the obesity associated BMAT and whether this is associated with increased bone quantity and quality (184). The study showed that BMAT was increased by 44% in diet-induced obesity measured by osmium-µCT, whereas exercise was associated with reduced BMAT (–48%), as well as increased trabecular bone volume (+19%), and higher bone stiffness in obese mice

The anti-diabetic drug, rosiglitazone, which is a PPARγ-agonist, is known to significantly increase BMA and fracture risk. It is shown that physical exercise significantly lowers BMA in rosiglitazone-treated male C57Bl/6J mice (185).

A randomized clinical trial investigated the effect of exercise on BMA in forty patients with chronic non-specific low back pain, and revealed that lumbar vertebral fat fraction was lower post exercise in these patients compared to the baseline (186). Chronic alcohol consumption is another risk factor in osteoporotic-bone loss associated with increased BMA (187). It is shown that 3 months of chronic alcohol consumption in the diet of 4-week-old male Sprague-Dawley rats leads to skeletal abnormalities together with increased BMA.

## Pharmacological treatment

Several drugs have been developed to improve bone parameters in metabolic bone diseases. Additional challenge in treatment of bone diseases is now to reduce BMAT volume, which may lead to increased bone mass and strength, together with decreased risk of bone fracture.

Several antidiabetic drugs have been tested in clinical settings for treatment of metabolic bone disease, including insulin-sensitizers such metformin, modified TZD analogs (PPARγ-independent), inhibitors of inflammatory molecules such as DPP4 (dipeptidyl peptidase-4). Ambrosi et al. has reported that DDP4 inhibition (secreted from adipocytes and increased with obesity in bones) in mouse model of obesity improved bone parameters and decreased BMAT (146). Similar results have been demonstrated in OVX female mice treated with DPP4 inhibitor (188). Clinical studies using DDP4 inhibitor, sitagliptin, showed improvement of bone turnover markers and decreased fracture risk in diabetic patients (189, 190).

Studies using metformin treatment in rodents and humans showed positive results or no changes in bone parameters suggesting that metformin might have a positive effect on bone metabolism in diabetic conditions, depending on the other medical complications associated with diabetes and length of disease manifestation. However, BMAT parameters were not evaluated in these conditions (191). Human studies of the Rochester cohort suggest that metformin decreases fracture risk in T2D patients (hazard ratio 0.7) (192). Although the ADOPT studies did not demonstrate beneficial effects of metformin on fracture risk (193), they showed decreased levels of bone resorption marker CTX and, contrary to the animal studies, decreased levels of bone formation marker P1NP (194). In a large case-control study metformin utilization was also associated with a reduction in the risk of fractures (195). In contrast, there are case control studies in which no association was observed between treatment with the insulin-sensitizing drug metformin and incidence of bone fractures in T2D patients (196).

In order to decrease the negative side effects of TZDs, novel TZD analogs with PPARγ independent effect on glucose metabolism have been developed. Stechschulte et al., reported that post-translational modifications of PPARγ at S112 and S273, which influence PPARγ pro-adipocytic and insulin sensitizing activities, improved bone parameters and decreased BMAT in lean and obese mice (197). Blocking PPARγ only at S273 by SR10171 had a beneficial effect on trabecular and cortical bone while maintaining its metabolic effect on glucose metabolism.

Another TZD analogue, with PPARγ independent affinity, MSDC-0602 has been reported to have a beneficial effect on bone parameters by decreasing osteoclast activation and BMAT formation in lean aged mice, while keeping its insulin sensitizing features (198). This drug is in clinical trial 2b (199).

Another category of pharmacological treatment used as anti-aging drugs has been tested to improve bone parameters and decreased bone fragility (e.g. resveratrol). Our recent study showed that resveratrol has anti-adipogenic effect along with decreased senescence in primary BMSCs isolated from patients with osteoporosis (34), which was also confirmed in animal studies with OVX rats (200, 201). However, the clinical relevance of these findings has not yet been investigated.

Sclerostin (SOST), osteocyte-secreted biomolecule with a negative impact on bone homeostasis (as it is an inhibitor of Wnt signaling), has become a potential drug target for anabolic treatment of diverse bone loss states (202). SOST circulating levels are increased with obesity, aging, and osteoporosis (203). Thus, neutralizing antibodies against sclerostin (SostAb) have been developed for testing its potential in clinical use to decrease bone fragility in osteoporosis and obesity. Using SostAb in animals showed improvement in fracture healing in diabetic mice (204) and OVX rats (205, 206). SostAb treatment in T2D rat models has also been shown to improve bone mass and strength (207). Clinical trials with SostAb have shown promising results with improvement of bone density and bone strength in postmenopausal women (208–210) Therefore, SostAb seems to be a promising osteoanabolic drug for treatment of skeletal fragility observed in osteoporosis, obesity and T2D (211). However, these clinical studies did not evaluate the effects of SostAb on BMAT. Recent studies in animal models suggest that SostAb may revert negative effect of rosiglitazone-induced increased BMAT in BM (212) and thus confirms its impact on adipogenesis of BMSCs (213).

### Antiresorptive treatments

Several antiresorptive agents including bisphosphonates, or RANKL antagonist, denosumab have been proven to safely reduce fracture risk in various high-risk populations (214, 215). They are recommended as first-line therapy for patients with osteoporosis. However, using antiresorptive drugs in treatment of osteoporosis in T2D patients might not be the perfect choice as patients with metabolic diseases (obesity, insulin resistance or T2D) have decreased bone turnover and their use would just further diminish the bone homeostasis. In addition, a recent clinical study showed that denosumab-treated patients had improved HbA1c similar to the effect of other anti-diabetic drugs, suggesting its possible insulin sensitizing effect (216). Clinical and animal studies have indicated beneficial effect of denosumab on insulin sensitivity, bone formation and muscle strength (217). However, its impact on BMAT volume has not been evaluated yet and more clinical investigations are needed to include this parameter to measure in their outcomes.

## Conclusion and future perspectives

**Figure 1:**
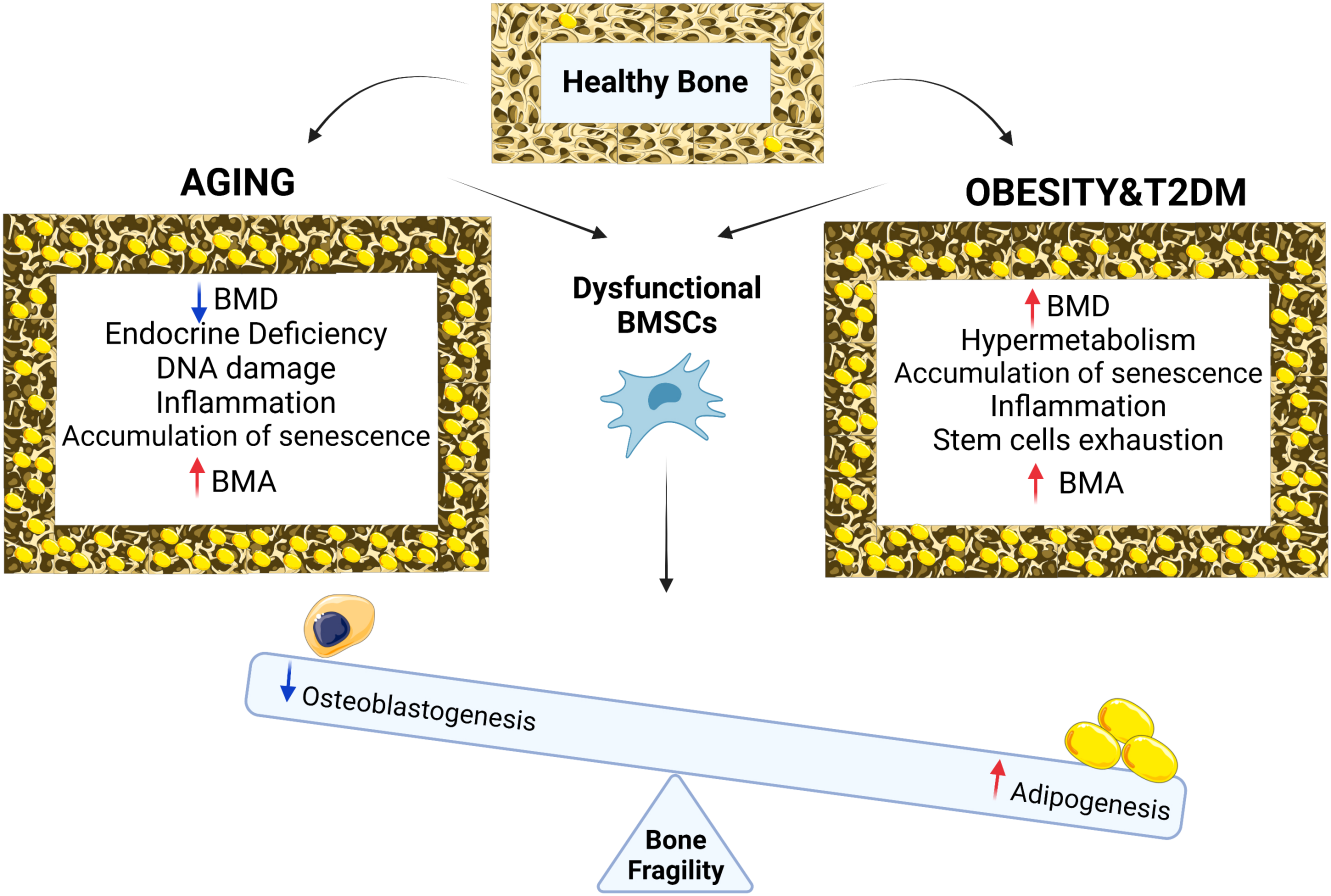
Chronological aging in bone is associated with reduction in BMD, endocrine deficiency, DNA damage, inflammation, accumulation of senescence, BMA and osteoporotic bone phenotype while with metabolic diseases such as obesity and type 2 diabetes, bone phenotype is associated with increased in BMD, cellular hypermetabolism, stem cell exhaustion, accumulation of senescence, inflammation, BMA and osteoporotic bone phenotype. Both conditions (aging vs metabolic diseases of obesity and T2D) result in BMSCs dysfunction leading to differentiation imbalance decreasing osteogenesis, increasing adipogenesis, BMA and bone fragility.

Increased accumulation of BMAT in bones has been recognized as a feature of aging bones associated with increased fracture risk. However, endocrine, and metabolic disturbances in organism such as hormone deficiency, obesity, and T2D can accelerate the detrimental changes in bone homeostasis and contribute to the early onset of osteoporosis and microstructural changes that compromise the bone strength (**Figure 1**.). Several mechanisms underlie these symptoms including transcription factors, DNA damage, ROS production, accumulation of senescent cells in BM microenvironment and secretion of bioactive molecules regulating function of BMSCs directing their fate towards BMAds. Using different animal models of osteoporosis and metabolic disorders, it has been shown that targeting senescent cells and regulation of hormonal levels can modulate the negative impact of expanded BMAT on bone loss fragility. More importantly, recent clinical interventions including patients with a broad range of age and complications reported that lifestyle modifications such as a special diet, caloric restriction, and physical activity may modulate BMAand improve bone parameters. However, more follow up studies are needed to evaluate the changes on bone structure and cellular modifications in real time at different timepoints, to establish the impact of such interventions on bone remodeling. Finally, including more imaging methods to evaluate bone and BMAT parameters in future clinical studies can facilitate further examination of the BMAT and its roles in skeletal fragility in the context of obesity and T2D in aging population, thereby providing novel therapeutic possibilities and also possibly, development of better approaches for estimation of fracture risk.

## Authors contributions

DA, MT, FF and AJ researched data, wrote and reviewed the manuscript. MK contributed to the conceptual framing of the manuscript. DA and AJ edited the manuscript. All authors contributed to the article and approved the submitted version

## Funding

(MT) START UP Research Program from IPHYS, the Czech Science Foundation GACR 20-03586S, GACR 22-12243S, EFSD/NovoNordisk foundation Future leaders award (NNF20SA0066174). (AJ, MK) Olav Thon Foundation (2019).

## Conflict of interest

The authors declare that the research was conducted in the absence of any commercial or financial relationships that could be construed as a potential conflict of interest.

## Publisher’s note

All claims expressed in this article are solely those of the authors and do not necessarily represent those of their affiliated organizations, or those of the publisher, the editors and the reviewers. Any product that may be evaluated in this article, or claim that may be made by its manufacturer, is not guaranteed or endorsed by the publisher.

## Abbreviations

BM, Bone Marrow; BMA, Bone marrow adiposity; BMAT, Bone marrow adipose tissue; BMD, Bone mineral density; BMSCS, Bone Marrow Stromal Stem Cells; BMPs, Bone morphogenic proteins; DEXA, Dual energy X-ray absorptiometry; HFD, High fat diet; IGF-1, Insulin like growth factor-1; IL-6, Interleukin 6; OVX, Ovariectomy; P1NP, Procollagen 1Intact N-Terminal Propeptide; PPARγ, Peroxisome proliferation-activated receptor gamma; RANKL, Receptor activator of nuclear factor kappa β ligand; RUNX2, Runt related transcription factor 2; SASP, Senescence associated secretory phenotype markers; TNFα, Tumor necrosis factor alpha.
